# Ultrafast dynamic contrast-enhanced breast MRI may generate prognostic imaging markers of breast cancer

**DOI:** 10.1186/s13058-020-01292-9

**Published:** 2020-05-28

**Authors:** Natsuko Onishi, Meredith Sadinski, Mary C. Hughes, Eun Sook Ko, Peter Gibbs, Katherine M. Gallagher, Maggie M. Fung, Theodore J. Hunt, Danny F. Martinez, Amita Shukla-Dave, Elizabeth A. Morris, Elizabeth J. Sutton

**Affiliations:** 1 Breast Imaging Service, Department of Radiology, Memorial Sloan Kettering Cancer Center, New York, NY USA; 2 Department of Medical Physics, Memorial Sloan Kettering Cancer Center, New York, NY USA; 3 GE Healthcare, New York, NY USA

**Keywords:** Breast carcinoma, Ultrafast dynamic contrast-enhanced magnetic resonance imaging, Maximum slope, Bolus arrival time, Molecular subtype, Invasive lobular carcinoma, Ductal carcinoma in situ

## Abstract

**Background:**

Ultrafast dynamic contrast-enhanced magnetic resonance imaging (DCE-MRI)-derived kinetic parameters have demonstrated at least equivalent accuracy to standard DCE-MRI in differentiating malignant from benign breast lesions. However, it is unclear if they have any efficacy as prognostic imaging markers. The aim of this study was to investigate the relationship between ultrafast DCE-MRI-derived kinetic parameters and breast cancer characteristics.

**Methods:**

Consecutive breast MRI examinations between February 2017 and January 2018 were retrospectively reviewed to determine those examinations that meet the following inclusion criteria: (1) BI-RADS 4–6 MRI performed on a 3T scanner with a 16-channel breast coil and (2) a hybrid clinical protocol with 15 phases of ultrafast DCE-MRI (temporal resolution of 2.7–4.6 s) followed by early and delayed phases of standard DCE-MRI. The study included 125 examinations with 142 biopsy-proven breast cancer lesions. Ultrafast DCE-MRI-derived kinetic parameters (maximum slope [MS] and bolus arrival time [BAT]) were calculated for the entire volume of each lesion. Comparisons of these parameters between different cancer characteristics were made using generalized estimating equations, accounting for the presence of multiple lesions per patient. All comparisons were exploratory and adjustment for multiple comparisons was not performed; *P* values < 0.05 were considered statistically significant.

**Results:**

Significantly larger MS and shorter BAT were observed for invasive carcinoma than ductal carcinoma in situ (DCIS) (*P* < 0.001 and *P* = 0.008, respectively). Significantly shorter BAT was observed for invasive carcinomas with more aggressive characteristics than those with less aggressive characteristics: grade 3 vs. grades 1–2 (*P* = 0.025), invasive ductal carcinoma vs. invasive lobular carcinoma (*P* = 0.002), and triple negative or HER2 type vs. luminal type (*P* < 0.001).

**Conclusions:**

Ultrafast DCE-MRI-derived parameters showed a strong relationship with some breast cancer characteristics, especially histopathology and molecular subtype.

## Introduction

Breast magnetic resonance imaging (MRI) is based on the dynamic contrast-enhanced (DCE) protocol with at least three phases: pre-contrast, early, and delayed phases. The phases are acquired with a scan time of 60–120 s per phase [1, 2] to capture the peak enhancement of breast cancer, which usually occurs within the first two minutes after contrast injection, with high enough spatial resolution [2–4].

Ultrafast DCE-MRI enables high temporal resolution (usually 4–8 s) while preserving high spatial resolution, usually using various acceleration methods, e.g., parallel imaging, view sharing, and compressed sensing. When employed in the very early phase (0–60 s after contrast injection), it can generate kinetic parameters reflecting contrast agent inflow effects. The diagnostic utility of these generated parameters in differentiating malignant from benign lesions and in improving positive predictive value has been proven in recent years [5–15]. Moreover, several studies have shown that these parameters present higher or comparable accuracy to Breast Imaging Reporting and Data System (BI-RADS) [1] delayed phase kinetic curve assessment, suggesting that ultrafast DCE-MRI in the very early phase may substitute for the standard DCE-MRI delayed phase [5, 7, 9, 13–15].

Besides their role as a diagnostic tool, ultrafast DCE-MRI parameters have the potential to serve as prognostic imaging markers. They may facilitate our understanding of intra- or inter- tumor heterogeneity. They may enable us to monitor intratumoral dynamics throughout the course of neoadjuvant therapy non-invasively without repeated biopsies. To further explore the ability of ultrafast DCE-MRI, in this study, we aimed to investigate the relationship between ultrafast DCE-MRI-derived kinetic parameters and breast cancer characteristics.

## Methods

### Patients and lesions

The institutional review board approved our retrospective study and waived the need for written informed consent. We conducted this study in compliance with the Health Insurance Portability and Accountability Act.

**Fig. 1:**
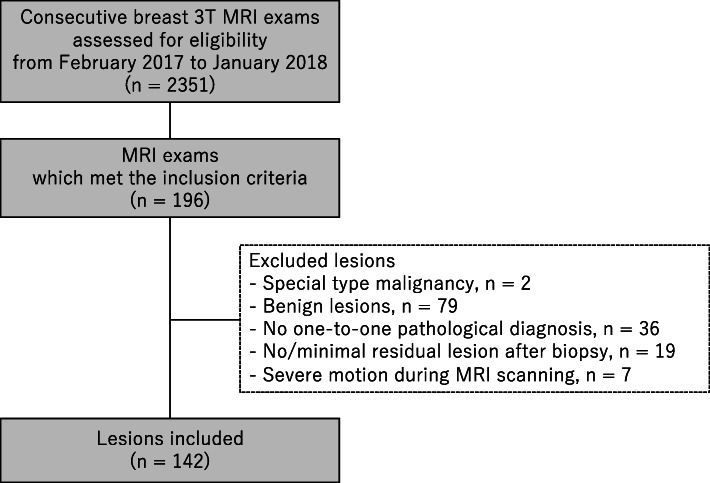
Flowchart of lesions

From February 2017 to January 2018, contrast-enhanced breast 3T MRI examinations routinely included ultrafast DCE-MRI along with standard DCE-MRI as part of a hybrid DCE-MRI protocol in our institution; however, clinical assessments including BI-RADS categorization were made using standard DCE-MRI without ultrafast DCE-MRI. We conducted a retrospective search of the institutional electronic medical record during this period to identify examinations that met the following inclusion criteria: (1) performed with a hybrid DCE-MRI protocol on a single 3T scanner with a 16-channel breast coil, (2) depicted pathologically proven breast lesions, (3) categorized as BI-RADS MRI 4–6, and (4) not performed for post-treatment evaluation after chemotherapy or surgery. Of 2,351 consecutive contrast-enhanced breast 3T MRI examinations including screening and diagnostic examinations, 196 examinations met the inclusion criteria. We included all pathologically proven breast lesions depicted on these examinations with the exception of the following lesions: lesions pathologically diagnosed as a special type malignancy (*n* = 2, malignant phyllodes tumor, 1; spindle cell sarcoma, 1), lesions pathologically diagnosed as benign lesions (*n* = 79), lesions without a one-to-one pathological diagnosis (*n* = 36), lesions with no or minimal residual enhancement difficult to differentiate from post-biopsy change (*n* = 19; invasive carcinoma, 15; ductal carcinoma in situ [DCIS], 4), and lesions with severe patient motion during MRI scanning which could not be resolved by motion correction technique (*n* = 7; invasive carcinoma, 6; DCIS, 1). In total, 125 examinations with 142 pathologically proven breast cancer lesions were included in this study (Fig. 1). The current study included a partial overlap in the study cohort, with separate studies investigating the diagnostic performance of ultrafast DCE-MRI-derived parameters [15] and the efficacy of radiomic analysis using standard DCE-MRI for sub-1 cm lesions [16]: 97 examinations with 106 lesions and 74 examinations with 79 lesions, respectively. Detailed information of the overlap is shown in the Supplemental Table.

### MRI

**Fig. 2:**
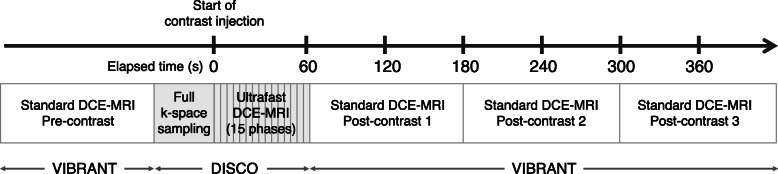
Hybrid protocol of ultrafast and standard dynamic contrast-enhanced magnetic resonance imaging (DCE-MRI)

All patients underwent MRI examinations in the prone position on a single scanner: 3.0T MRI system (Discovery 750, GE Medical Systems, Waukesha, WI) with a dedicated 16-channel breast coil (Sentinelle, Invivo, Gainesville, FL). We used the same MRI protocol as in a previous study [15]. Briefly, one pre-contrast phase and three post-contrast phases of standard DCE-MRI, and 15 phases of ultrafast DCE-MRI were acquired. After pre-contrast imaging was acquired for standard DCE-MRI, ultrafast DCE-MRI was acquired continuously for 15 timepoints during the first approximately 60 s, starting simultaneously with the start of contrast injection. The contrast agent (Gadavist; Bayer Healthcare Pharmaceuticals Inc., Whippany, NJ) was administered at a concentration of 0.1 mmol gadobutrol per kg body weight and a rate of 2 ml/s, followed by a 40 ml saline flush at the same rate. Immediately after ultrafast DCE-MRI, standard DCE-MRI was acquired continuously at three timepoints (Fig. 2). The acquisition parameters were as follows: for ultrafast DCE-MRI using a 3D fat-suppressed T1-weighted differential sub-sampling with cartesian ordering (DISCO) sequence, TR = 3.8 ms, TE1 and TE2 = 1.1/2.2 ms, flip angle = 12°, field of view = 34 cm × 34 cm, acquired matrix = 212 × 212, in-plane spatial resolution = 1.6 × 1.6 mm, thickness = 1.6 mm, number of slices = 166 (or higher to ensure full breast coverage), bandwidth = ± 142.86 kHz, ARC acceleration factor = 4 (phase) × 2 (slice), temporal resolution = 2.7–4.6 s/phase, temporal foot-print (time to acquire a complete k-space for a phase) = 9 s, axial orientation. For standard DCE-MRI using a 3D fat-suppressed T1-weighted volume imaging breast assessment (VIBRANT) sequence, TR/TE = 7.9/4.3, flip angle = 12°, field of view = 34 cm × 34 cm, acquired matrix = 300 × 300, in-plane spatial resolution = 1.1 × 1.1 mm, adiabatic fat suppression, number of slices = 190 (depending on coverage), bandwidth = ± 62.5 kHz, ASSET acceleration factor = 2 (phase) × 1 (slice), scan time = ~ 120 s/phase, axial orientation.

### Image interpretation

Radiologists 1 and 2 (NO and ESK with 7 and 13 years of experience in breast MRI, respectively) reviewed the first post-contrast standard DCE-MR images for all lesions and evaluated the BI-RADS MRI lesion type (mass, non-mass enhancement [NME], and focus) in consensus. The radiologists were blinded to pathological reports.

### Image analysis

#### Segmentation

Radiologist 1 (NO) used the GenIQ software (GE Healthcare, Waukesha, WI) to segment the lesions. Semi-automatic volumetric segmentation was performed on the first post-contrast image of the standard DCE-MRI, and the segmentation was thereafter cloned to all other phases of ultrafast and standard DCE-MRI. First, the radiologist performed a manual segmentation on a single slice. Second, the software yielded volumetric segmentation based on the single slice segmentation. Finally, the radiologist manually modified the volumetric segmentation, if necessary. The radiologist examined the time-signal intensity curves of the segmented volumes and ultrafast DCE-MR images, and a localized, rigid motion correction technique was applied if patient motion was apparent on imaging.

#### Parameter calculation

Based on the volumetric segmentation, the same software calculated heuristic kinetic parameters from ultrafast DCE-MRI. In the software, data at the first three timepoints were considered as baseline (theoretically pre-contrast) and signal intensity was automatically converted to contrast agent concentration using Eqs. (1) and (2) as reported by Li et al. [17]. Primarily, the software computed the T1 values in the tissue during passage of the contrast (T1_post_), ignoring the T2* and B1 non-uniformity effects. The baseline signal was computed using the signal values before the bolus arrival time. The pre-contrast baseline T1 (T1_pre_) value for breast tissue was fixed at 1444 ms for 3T, based on Rakow-Penner et al. [18]. A system of equations for signal intensity (using Eq. (1) in Li et.al [17].) was set up for baseline and post contrast intensity changes, and then re-arranged for computing the T1_post_ using the knowledge of S_pre_, S_post_, flip angle, and T1_pre_. Equation (2) from Li et.al [17] then allowed for computing contrast agent concentration using T1_pre_ and T1_post_ and relaxivity of the gadolinium (fixed in GenIQ software to 4.9 s^−1^ mM^−1^).

For each lesion, the following parameters derived by GenIQ were measured:

- Maximum slope [MS] (mmol/s) = the slope of the steepest part of the concentration uptake curve (between the bolus arrival time and the peak arrival time)
- Bolus arrival time [BAT] (s) = the time from start of contrast injection to tracer bolus arrival at a lesion

BAT was calculated by GenIQ software using the gradient based method described in the literature by Mehrtash et al. [19]: the concentration curve was first transformed into the logarithmic domain for noise suppression and values < 0.001 mmol/l were set to zero. For each pixel, the peak timepoint in the concentration curve was computed, and the software performed a backward search for the BAT. The BAT was determined as the timepoint where the concentration curve changes direction, from steep descent to gradual flat roll-off from the point of backward search point. The BAT measured in our study was the average BAT of all the pixels with the tumor ROI.

### Cancer characteristics

Histopathology was retrospectively determined; a careful review of pathological reports of both core biopsy specimens and surgical specimens was performed to acquire one-to-one pathological diagnoses of the lesions depicted on MRI. All lesions were classified into invasive carcinoma or DCIS. Microinvasive carcinomas were classified as DCIS because they consist primarily of DCIS. Invasive carcinomas were further classified based on their tumor grade (grade 1, 2, or 3), their histopathological subtype (invasive ductal carcinoma [IDC], invasive lobular carcinoma [ILC], or mixed invasive ductal/lobular carcinoma [mixed IDC/ILC]), and their molecular subtype (triple negative type [hormone receptor negative, HER2 negative], HER2 type [HER2 positive regardless of the hormone receptor positivity/negativity], or luminal type [hormone receptor positive, HER2 negative]). Positive lymph node metastasis was pathologically determined by reviewing reports of either fine needle biopsy, sentinel lymph node biopsy, or axillary dissection. Oncotype DX recurrence score (Genomic Health, Redwood City, CA) was available if the lesion was diagnosed as luminal type, T1–2, and N0–N1mi (micrometastases) on surgical pathology. The recurrence risk was evaluated based on the score: low recurrence risk (score ≤ 17), intermediate recurrence risk (18 ≤ score ≤ 30), and high recurrence risk (score ≥ 31).

### Statistical analysis

Statistical analyses were performed using JMP® Version 10.0.2 (SAS Institute Inc., Cary, NC) and R Version 3.5.1 (R Foundation for Statistical Computing, Vienna, Austria). All comparisons were exploratory and adjustment for multiple comparisons was not performed; *P* values < 0.05 were considered statistically significant.

To assess the inter-reader reliability of MS and BAT, intraclass correlation coefficient was assessed using a cohort of 24 lesions randomly derived from the whole cohort, of which radiologist 3 (MCH, 10 years of experience in breast MRI) independently performed segmentation and calculated each parameter using the same method as radiologist 1. The intraclass correlation coefficient between the two radiologists was evaluated following the interpretation proposed by Cicchetti [20]: a value of 0.75–1.00 was considered excellent agreement; 0.60–0.74, good agreement; 0.40–0.59, fair agreement; and less than 0.40, poor agreement.

To test the hypothesis that MS and BAT may differ between invasive carcinoma and DCIS, we used generalized estimating equations (GEE) in R, accounting for the presence of multiple lesions per patient. The maximum cluster size present was three. The cluster size was effectively the number of lesions per patient. In invasive carcinomas, to test the hypothesis that MS and BAT may differ in invasive carcinomas according to cancer characteristics (tumor grade, histopathology, molecular subtype, lymph node metastasis status, and recurrence risk), we divided the invasive carcinomas into the respective two subgroups and compared MS and BAT between groups using GEE. For the cancer characteristics in which both MS and BAT were significantly different, we also performed exploratory multivariate logistic regression modeling using GEE. Area under the ROC curve (AUC) of the models was compared using the DeLong’s test [21].

## Results

### Patients

In total, 142 pathologically proven breast cancer lesions in 125 female patients (mean age, 49.6 years; SD, 11.9 years; range, 21–79 years) were analyzed. Of the 125 patients, 111 had a single lesion, 11 had double lesions, and 3 had triple lesions. Four patients had bilateral cancers. Table 1 summarizes the detailed information of the patients including age, menopausal status, BI-RADS category, past history of breast cancer, and family history of breast or ovarian cancer. The median interval between breast MR imaging and core biopsy was 14.6 days (interquartile range of 8.6–21.8 days).

**Table 1:** Detailed information of patients

Patient characteristics	Total (*n* = 125)
Age	
Mean ± SD	49.6 ± 11.9 years
Range	21–79 years
Menopausal status	
Pre-menopause	78 (62)
Post-menopause	47 (37)
BI-RADS	
Category 6	68 (54)
Category 5	1 (1)
Category 4	56 (44)
Past history of breast cancer	
Positive	5 (4)
Negative	120 (95)
Family history of breast cancer	
Positive	66 (52)
Negative	57 (45)
Not available	2 (2)
Family history of ovarian cancer	
Positive	12 (10)
Negative	111 (88)
Not available	2 (2)

Unless otherwise specified, data represent the number of patients and data in parentheses are percentages

*SD* standard deviation

### Cancer characteristics

The 142 lesions comprised 96 mass (68%), 44 NME (31%), and 2 focus (1%) lesions. They were pathologically diagnosed as 124 invasive carcinomas (87%) and 18 DCIS (13%). The median size of the invasive carcinomas was 19.5 mm (interquartile range of 13–32.3 mm), and the median size of the DCIS was 13.5 mm (interquartile range of 9.3–19.5 mm). Table 2 summarizes the detailed information of the cancer characteristics including lesion type, lesion size, tumor grade, histopathology, molecular subtype, lymph node metastasis status, and Oncotype DX recurrence score.

**Table 2:** Cancer characteristics

		Invasive carcinoma (***n*** = 124)	DCIS (***n*** = 18)
**Lesion type**			
Mass		91 (73)	5 (28)
Non-mass enhancement		31 (25)	13 (72)
Focus		2 (2)	0 (0)
**Lesion size**			
Median		19.5 mm	13.5 mm
1st quartile, 3rd quartile		13 mm, 32.3 mm	9.3 mm, 19.5 mm
**Tumor grade of DCIS**			
Microinvasive carcinoma		NA	3 (17)
High grade DCIS		NA	4 (22)
Intermediate grade DCIS		NA	10 (56)
Low grade DCIS		NA	1 (6)
Tumor grade of invasive carcinoma*			
Grade 1 (well differentiated)		7 (6)	NA
Grade 2 (moderately differentiated)		48 (39)	NA
Grade 3 (poorly differentiated)		56 (45)	NA
Not available		13 (10)	NA
Histopathology*			
Invasive ductal carcinoma		98 (79)	NA
Invasive lobular carcinoma		20 (16)	NA
Mixed invasive ductal/lobular carcinoma		6 (5)	NA
Molecular subtype*			
Triple negative type	(HR−, HER2−)	12 (10)	NA
HER2 type (HR−)	(HR−, HER2+)	5 (4)	NA
HER2 type (HR+)	(HR+, HER2+)	16 (13)	NA
Luminal type	(HR+, HER2−)	91 (73)	NA
Lymph node metastasis status*			
Positive		57 (46)	NA
Negative		64 (52)	NA
Not available		3 (2)	NA
**Oncotype DX recurrence score**^**†**^			
High risk		2 (4)	NA
Intermediate risk		13 (29)	NA
Low risk		30 (67)	NA

*Data for invasive carcinomas

^†^Data for 45 invasive carcinomas for which Oncotype DX score was available

*DCIS* ductal carcinoma in situ, *NA* not applicable

### Inter-reader reliability

The inter-reader reliability was excellent for MS with an inter-reader intraclass correlation coefficient of 0.951 (95% CI 0.891, 0.978) and excellent for BAT with an inter-reader intraclass correlation coefficient of 0.948 (95% CI 0.885, 0.977).

### Invasive carcinoma vs. DCIS

**Fig. 3:**
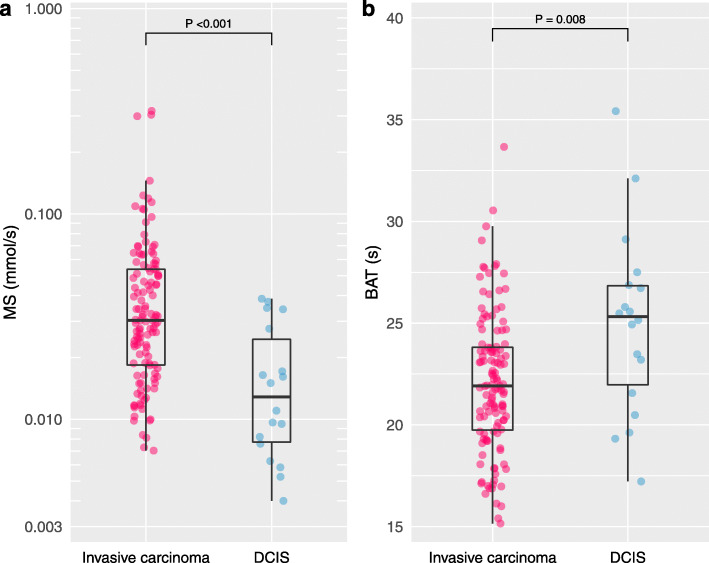
Maximum slope and bolus arrival time for invasive carcinoma and DCIS. Invasive carcinomas presented significantly larger maximum slope (MS) and shorter bolus arrival time (BAT) than DCIS (*P* < 0.001 and *P* = 0.008, respectively)

Invasive carcinomas showed significantly larger MS and shorter BAT than DCIS (*P* < 0.001 and *P* = 0.008, respectively). Table 3 summarizes these results and Fig. 3 shows the box and whisker plots.

**Table 3:** Maximum slope and bolus arrival time according to histopathology

	No. of lesions^**†**^	Maximum slope (mmol/s)			Bolus arrival time (s)		
		Median	IQR	*P* value	Median	IQR	*P* value
**Histopathology**				< 0.001*			0.008*
**Invasive carcinoma**	124 (87)	0.030	0.018–0.054		21.9	19.7–23.8	
**DCIS**	18 (13)	0.013	0.007–0.029		25.3	21.3–27.0	

*DCIS* ductal carcinoma in situ, *IQR* interquartile range

*P < 0.05

^†^Data in parentheses are percentages

### Invasive carcinomas

**Fig. 4:**
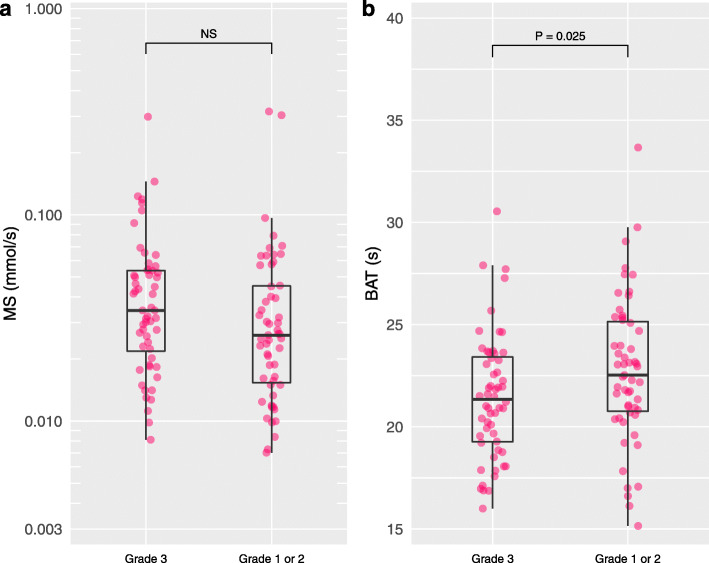
Maximum slope and bolus arrival time for tumor grade 3 and tumor grades 1–2. Tumor grade 3 presented significantly shorter bolus arrival time (BAT) than tumor grades 1–2 (*P* = 0.025)

**Fig. 5:**
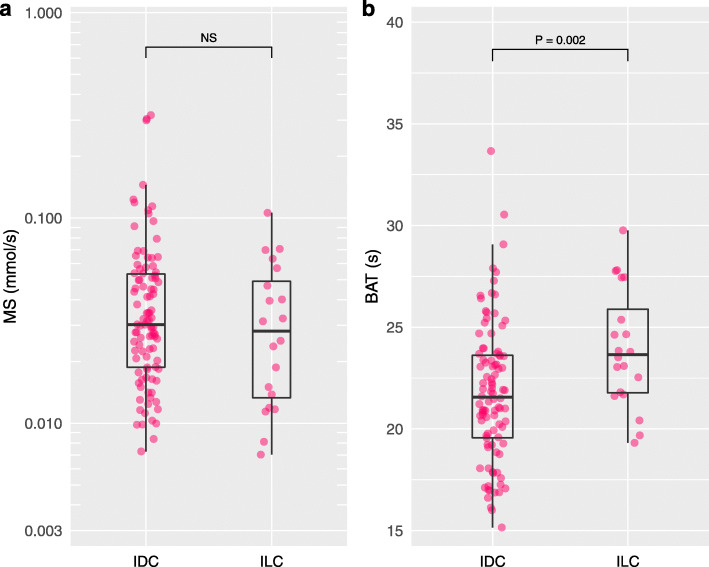
Maximum slope and bolus arrival time for invasive ductal carcinoma and invasive lobular carcinoma. Invasive ductal carcinoma (IDC) presented significantly shorter bolus arrival time (BAT) than invasive lobular carcinoma (ILC) (*P* = 0.002)

**Fig. 6:**
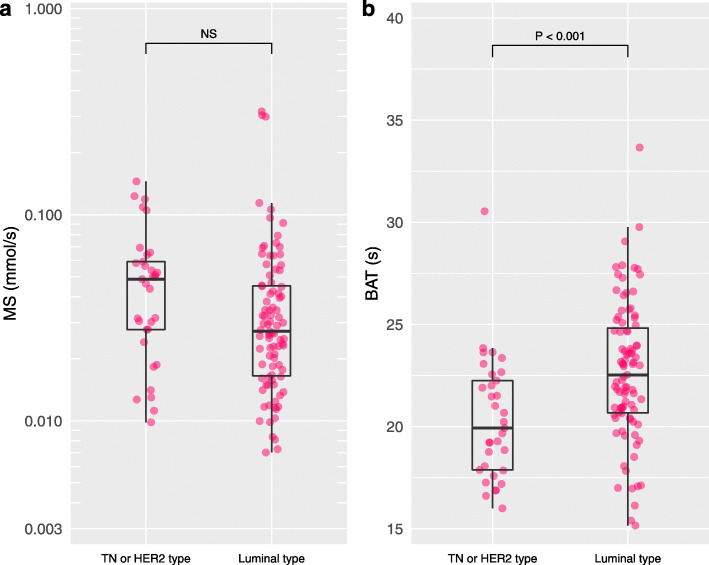
Maximum slope and bolus arrival time for triple negative or HER2 type and luminal type. Triple negative (TN) or HER2 type presented shorter bolus arrival time (BAT) than luminal type (*P* < 0.001)

**Fig. 7:**
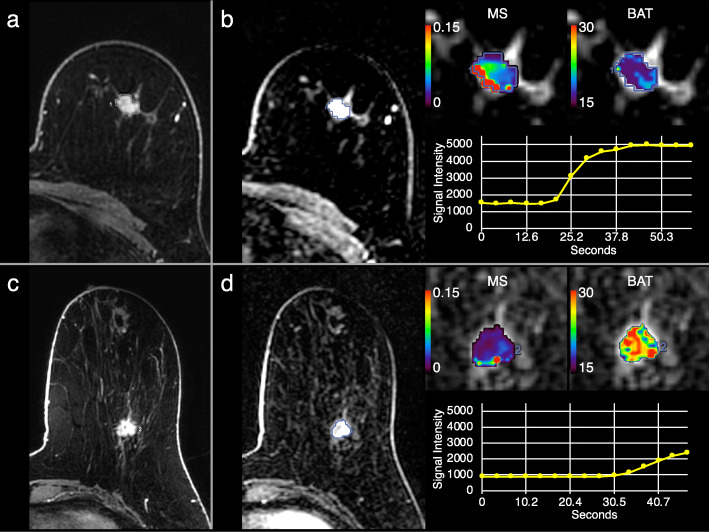
Dynamic contrast-enhanced magnetic resonance images (DCE-MRI) and parametric maps of representative cases. **a**, **b** Triple negative type invasive ductal carcinoma (IDC) in the left breast of a 55-year-old woman. **c**, **d** Luminal type IDC in left breast of a 54-year-old woman. Standard DCE-MRI (first post-contrast phase; **a**, **c**) and ultrafast DCE-MRI (15th phase, generated parametric maps, and signal intensity curves; **b**, **d**) are shown. The triple negative type IDC showed rapid increase of signal intensity with maximum slope (MS) of 0.145 mmol/s and bolus arrival time (BAT) of 18.8 s. The luminal type IDC showed slow increase of signal intensity with MS of 0.054 mmol/s and BAT of 27.7 s

Tumor grade 3 presented significantly shorter BAT than tumor grades 1–2 (*P* = 0.025) (Fig. 4). IDC presented significantly shorter BAT than ILC (*P* = 0.002) (Fig. 5). Triple negative or HER2 type presented significantly shorter BAT than luminal type (*P* < 0.001) (Fig. 6). Figure 7 shows images of representative cases. Neither of the parameters were significantly different between lymph node metastasis positive lesions and lymph node metastasis negative lesions or between lesions with a high/intermediate recurrence risk and lesions with a low recurrence risk. Table 4 summarizes these results.

**Table 4:** Maximum slope and bolus arrival time according to invasive cancer characteristics

	No. of lesions^†^	Maximum slope (mmol/s)			Bolus arrival time (s)		
		Median	IQR	*P* value	Median	IQR	*P* value
Tumor grade	111			0.552			0.025*
Grade 3	56 (50)	0.034	0.022–0.054		21.3	19.3–23.4	
Grade 1 or 2	55 (50)	0.026	0.015–0.045		22.5	20.8–25.2	
Histopathology	118			0.133			0.002*
IDC	98 (83)	0.030	0.019–0.054		21.6	19.6–23.6	
ILC	20 (17)	0.028	0.013–0.049		23.7	21.8–25.9	
Molecular subtype	124			0.361			< 0.001*
HER2+ type or triple negative type	33 (27)	0.049	0.028–0.059		19.9	17.9–22.3	
Luminal type	91 (73)	0.027	0.017–0.045		22.5	20.7–24.8	
Lymph node metastasis status	121			0.467			0.714
Positive	57 (47)	0.030	0.016–0.051		21.9	20.2–23.8	
Negative	64 (53)	0.030	0.022–0.055		21.9	19.7–23.8	
Oncotype DX recurrence score	45			0.538			0.633
High/intermediate risk	15 (33)	0.032	0.025–0.049		22.2	20.2–24.7	
Low risk	30 (67)	0.026	0.015–0.046		21.7	20.4–23.7	

*MS* maximum slope, *BAT* bolus arrival time, *IQR* interquartile range, *IDC* invasive ductal carcinoma, *ILC* invasive lobular carcinoma

*P < 0.05

^†^Data in parentheses are percentages

‡Molecular subtype are classified as follows: triple negative type [hormone receptor negative, HER2 negative], HER2 type [HER2 positive regardless of the hormone receptor positivity/negativity], or luminal type [hormone receptor positive, HER2 negative]

### Exploratory multivariate logistic regression modeling

**Fig. 8:**
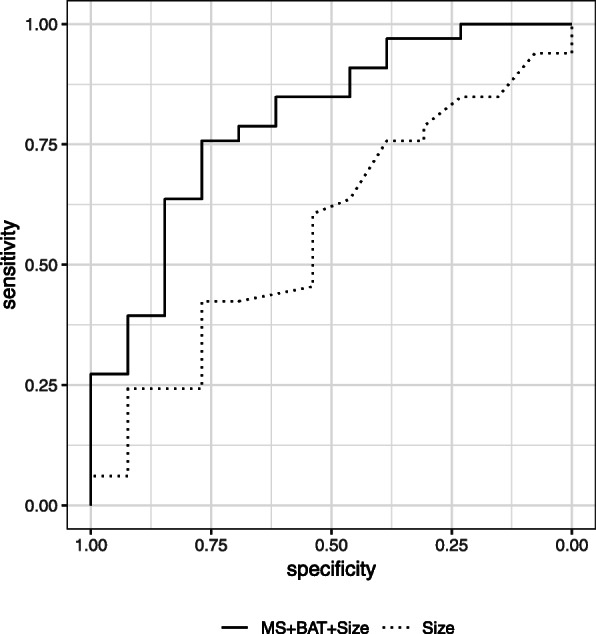
ROC curves of the logistic regression models for differentiating invasive carcinoma from DCIS. The AUC of the MS + BAT + size model was significantly higher than that of the size alone model: 0.80 vs. 0.56, *P* = 0.024

We performed an exploratory multivariate logistic regression modeling for differentiating invasive carcinoma from DCIS in NME lesions (Table 5). We selected MS, BAT, and size as variables. Of the three variables, only BAT was significantly predictive of invasive carcinoma (*P* = 0.022). The AUC of the MS + BAT + size model was significantly higher than that of the size alone model: 0.80 vs. 0.56, *P* = 0.024 (Fig. 8).

**Table 5:** Multivariate logistic regression model for differentiating invasive carcinoma from DCIS in non-mass enhancement lesions

Variables	Beta coefficient	***P*** value
**Maximum slope (mmol/s)**	29.30	0.154
**Bolus arrival time (s)**	− 0.22	0.022*
**Size (mm)**	0.03	0.527

*P < 0.05

## Discussion

We investigated ultrafast DCE-MRI, focusing on its relationship with breast cancer characteristics; previously, this topic has not been well investigated in the literature. Two kinetic parameters derived from ultrafast DCE-MRI showed a strong relationship with histopathology and molecular subtype. Specifically, we observed a significantly larger maximum slope (MS) and a shorter bolus arrival time (BAT) for invasive carcinoma compared with DCIS. Invasive cancers with more aggressive characteristics showed significantly shorter BAT compared with those with less aggressive characteristics: tumor grade 3 vs. tumor grades 1–2, IDC vs. ILC, and triple negative or HER2 type vs. luminal type. The above results suggest the ability of these parameters to serve as prognostic imaging markers. Although biomarkers from tissue samples are the standard practice for breast cancer management at this time, the two prognostic imaging markers may facilitate our understanding of tumor biology because MRI can capture “live” tumor non-invasively. They will be valuable especially for situations where surgery or repeated biopsy is not preferable, e.g., monitoring during the course of neoadjuvant therapy. For example, the two prognostic imaging markers may help us understand how tumor dynamics change throughout the course of neoadjuvant treatment, how the change differs according to tumor heterogeneity, etc. The two prognostic imaging markers are worthy of further investigation.

It has been suggested that the growth of DCIS might be associated with tumor-derived angiogenesis evidenced by increased microvessel density [22, 23]; previous studies have proven the presence of intense angiogenesis surrounding DCIS as well as the associated contrast enhancement [24, 25]. In agreement with previous studies reporting that DCIS present with a slower increase of enhancement than typical invasive carcinoma in standard DCE-MRI [26, 27], in our study, MS and BAT showed a significant difference between DCIS and invasive carcinoma. These results are in line with a previous study on microvessel density, in which the onset of angiogenic “switch” was shown to occur at the onset of hyperplasia and the greatest increase of angiogenesis was shown to occur with the process of invasion [28]. In exploratory multivariate logistic regression modeling in NME lesions, the MS + BAT + size model showed significantly higher AUC than the size alone model. Although further validation in larger sample size is necessary, this result may highlight the possible usage of the two prognostic imaging markers in the clinical setting where differential diagnosis of NME lesions (whether DCIS or invasive carcinoma) is challenging.

Invasive cancers with more aggressive characteristics showed significantly shorter BAT compared with those with less aggressive characteristics. These results agree with a previous study on ultrafast DCE-MRI that showed shorter time to enhancement for higher grade, hormone receptor negative, Ki-67 > 20% invasive cancers [12]. These results are also in line with previous studies in standard DCE-MRI that showed shorter time to enhancement for hormone receptor negative cancers [29] and slower increase of enhancement in ILC compared to IDC [30, 31]. As opposed to the above results, MS did not show a statistical difference, although the median MS tended to be larger for invasive cancers with more aggressive characteristics. In previous studies, MS in ultrafast DCE-MRI and early strong enhancement in standard DCE-MRI were reported to significantly correlate with high histological grade and hormone receptor negativity [12, 29]. Although there is no reliable way to explain the difference with previous results, technical differences in our calculation method and sampling bias might have affected our results. Further analyses are necessary to draw a more reliable conclusion.

The concentration curve of our ultrafast DCE-MRI protocol with 2.7–4.6 s of temporal resolution likely reflects early leakage of contrast agent from the vessels into the extravascular extracellular space rather than a pure perfusion effect [5, 32, 33]. Thus, MS may reflect abnormality and leakiness of vasculature associated with angiogenesis [34]. BAT may reflect flow resistance in tumor vessels as well as tumor metabolism associated with tumor angiogenesis; on breast MRI, increased breast vascularity associated with ipsilateral cancer [35, 36] and a shorter time interval between arterial and venous visualization for breast vessels with ipsilateral cancer have been observed [10]. In this study, we calculated BAT as the time from the start of contrast injection to the contrast arrival at a lesion, while the majority of previous studies calculated time to enhancement or time of arrival as the time from the start of the cardiac or aortic enhancement to the contrast arrival at a lesion because they regarded it important to consider the individual difference in heart cardiac function [7, 8, 12, 13]. This was because of the limitations of our software used for the parameter calculation. Conversely, the significant difference of BAT observed in some cancer characteristics may suggest that the parameter is somewhat robust in the context of the individual difference in cardiac function.

One consideration for this ultrafast DCE-MRI sequence is that it acquires the center of k-space more frequently than the outer k-space and uses a view-sharing technique to reconstruct the data at high temporal resolution. The outer k-space is shared between multiple phases, and hence the “temporal footprint” of each phase (the amount of time to collect the complete k-space for one phase) is longer than the reconstructed temporal resolution (in our case, the temporal footprint is 9 s, versus the temporal resolution of 2.7–4.6 s). As a result, there will be some temporal blurring depending on the size/shape of the features. In our protocol, we tried to minimize the temporal footprint by using a very aggressive acceleration factor (4 × 2), and so we were able to maintain a reasonable temporal footprint. While the downside of such an aggressive acceleration factor is a reduction in the signal-to-noise ratio, in this study, we aimed to acquire the kinetic information, and therefore, we prioritized the temporal resolution over the signal-to-noise ratio. We also optimized the phase direction to be left/right, to align with the direction of maximal coil separation, so we can maximize the acceleration factor in the phase direction without incurring too much aliasing artifacts.

Another consideration is that we used a dual echo DIXON technique because of its advantage in providing uniform fat suppression, which is sometimes difficult to achieve in the breast using standard fat suppression pulses. The downside is that there would be some T2* decay between the two echoes in Dixon acquisition, which may cause inaccurate signal intensity in the water image if the TE is long. In our acquisition, we minimized this effect by using the first in-phase and out-of-phase echo time (1.1 ms and 2.2 ms) [37], and since the breast does not usually have high T2* content (e.g., iron), the T2* decay effect was minimal. Also, in this study, we focused on the relative signal intensity changes instead of quantitative values like Ktrans, so the T2* decay effect would not affect the MS and BAT measurements.

MS and BAT were reproducible and feasible to generate using the clinically scanned MR images, thus demonstrating the clinical relevance of these kinetic parameters and empowering the incorporation of ultrafast DCE-MRI into standard clinical protocol. We note, moreover, that ultrafast DCE-MRI may have good compatibility with an abbreviated breast MRI protocol, which is a shortened screening protocol that acquires only the pre-contrast and first post-contrast images in the early phase [38], because it can generate kinetic information with higher or comparable diagnostic accuracy to BI-RADS delayed phase kinetic assessment [5, 7, 9, 13–15] without extending the abbreviated total scan time. The hybridization of these protocols may generate better screening protocols with more efficient throughput.

The present study has limitations. First, it was a retrospective study of a patient population consisting of both diagnostic and screening examination. In addition, we included post-biopsy lesions (BI-RADS 6 lesions), of which post-biopsy change or artifacts due to a biopsy clip could have affected the calculation of MS and BAT. Second, the lesion size and lesion type (mass, NME, and focus) and histopathology were not controlled. Oncotype DX scores were only available for half of the luminal type invasive carcinoma, which could have biased the results. These were the limitations due to the limited number of lesions available from patients with clinically scanned MRIs. Third, it was performed using data from a single institution and MRI was performed using a 3T magnet scanner and a 16-channel coil. We need further validation using different protocols, vendors, magnets, and coils. We also need to clarify the similarity/difference between software for parameter calculation.

## Conclusions

In conclusion, we demonstrate a significant relationship between ultrafast DCE-MRI-derived parameters and breast cancer characteristics. Although further research with larger cohort in multiple institutions will be needed to validate our study, ultrafast DCE-MRI may lead to further improvement of the breast MRI protocol by generating prognostic imaging markers of breast cancer.

## Supplementary information

**Additional file 1 :** Supplemental Table. Patient overlap with previous studies.

## Data Availability

The datasets used and/or analyzed during the current study are available from the corresponding author on reasonable request.

## Abbreviations

AUC: Area under the ROC curve
BAT: Bolus arrival time
BI-RADS: Breast Imaging Reporting and Data System
DCE: Dynamic contrast-enhanced
DCIS: Ductal carcinoma in situ
IDC: Invasive ductal carcinoma
ILC: Invasive lobular carcinoma
MRI: Magnetic resonance imaging
MS: Maximum slope
NME: Non-mass enhancement
TN: Triple negative
