# Supplementary material for: Ultrafast dynamic contrast-enhanced breast MRI may generate prognostic imaging markers of breast cancer
Source: Breast Cancer Res. 2020 May 28;22:58. doi: 10.1186/s13058-020-01292-9 (PMC7254650; doi:10.1186/s13058-020-01292-9)
Supplement: Supplementary file 1 — Additional file 1 : Supplemental Table. Patient overlap with previous studies. [file 13058_2020_1292_MOESM1_ESM.docx]

**Supplemental Table**

The current study included a partial overlap in the study cohort, with separate studies investigating the diagnostic performance of ultrafast DCE-MRI derived parameters (Reference 15) and the efficacy of radiomic analysis using standard DCE-MRI for sub-1 cm lesions (Reference 16).

Reference 15 aimed to investigate the diagnostic performance (i.e. malignant vs. benign) of ultrafast DCE parameters in sub1cm lesions. The current study aims to investigate the relationship between ultrafast DCE parameters and breast cancer characteristic (pathology, molecular subtype, lymph node metastasis status, oncotype DX). The aims differed between the studies. No. of the overlapping patient is 97 and No. of the overlapping lesions is 106.

Reference 16 did not use ultrafast DCE MRI data but standard DCE-MRI for radiomics analysis. The current study does not use standard DCE-MRI data but ultrafast DCE MRI. The used MRI sequences differed between the studies. No. of the overlapping patient is 54 +20 = 74, and No. of the overlapping lesions is 59+20 = 79.
